# aSi-EPID transit signal calibration for dynamic beams: a needful step for the IMRT in vivo dosimetry

**DOI:** 10.1007/s11517-013-1094-x

**Published:** 2013-07-09

**Authors:** Francesca Greco, Angelo Piermattei, Luigi Azario, Lorenzo Placidi, Savino Cilla, Rocchina Caivano, Vincenzo Fusco, Andrea Fidanzio

**Affiliations:** 1 Istituto di Fisica, Università Cattolica del Sacro Cuore, Largo F. Vito 1, 00168 Rome, Italy; 2 U.O.C di Fisica Sanitaria, Università Cattolica del Sacro Cuore, Rome, Italy; 3 U.O di Fisica Sanitaria, Fondazione di Ricerca e Cura Giovanni Paolo II, Campobasso, Italy; 4 Unità Operativa di Radioterapia e Fisica Sanitaria, Centro di Riferimento Oncologico della Basilicata, IRCCS, Rionero, PZ Italy; 5 Istituto Nazionale di Fisica Nucleare (INFN), Sezione di Roma Tre, Rome, Italy

**Keywords:** In vivo dosimetry, Electronic portal imaging device, IMRT dynamic

## Abstract

This work reports a method based on correlation functions to convert EPID transit signals into in vivo dose values at the isocenter point, *D*_iso_, of dynamic IMRT beams supplied by Varian linac. Dose reconstruction for intensity-modulated beams required significant corrections of EPID response, due to the X-ray component transmitted through multileaf collimator. The algorithm was formulated using a set of simulated IMRT beams. The beams were parameterized by means of a fluence inhomogeneity index, FI, introduced to describe the degree of beam modulation with respect to open beams. This way, all dosimetric parameters involved in *D*_iso_ reconstruction algorithm, such as the correlation functions, the correction factor for EPID to phantom distance and the modulated tissue maximum ratios, were determined as a function of the FI index. Clinical IMRT beams were used to irradiate a homogeneous phantom, and for each beam, the agreement between the reconstructed dose, *D*_iso_, and the dose computed by TPS, *D*_iso,TPS_, was well within 5 %. Moreover, the average ratios, *R*, between the *D*_iso_, and *D*_iso,TPS_, resulted equal to 1.002 ± 0.030. Thirty-five IMRT fields of 5 different patients undergoing radiotherapy for head–neck tumors were tested and the results were displayed on a computer screen after 2 min from the end of the treatment. However, 350 in vivo tests supplied an average ratio *R* equal to 1.004 ± 0.040. The in vivo dosimetry procedure here presented is among the objectives of a National Project financially supported by the Istituto Nazionale di Fisica Nucleare for the development of in vivo dosimetry procedures (Piermattei et al. in Nucl Instrum Methods Phys Res B 274:42–50, 2012) connected to the Record–Verify system of the radiotherapy center.

## Introduction

As pointed out by the Radiotherapy Risk Profile report by WHO [25] the major causes of severe radiotherapy incidents are due to human errors. Moreover, an IAEA review [10] reports that in vivo dosimetry (IVD) is an efficient method to trace dose delivery errors either during treatment planning or during the treatment delivery [15, 27]. This way, because of complexity of the radiotherapy techniques, IVD is becoming mandatory in some countries [16, 27].

The use of EPIDs to reconstruct the dose delivered to the patient is very promising, and at moment, the literature reports experiences for dose reconstruction in a single point, in many points on planes or in 3D [18, 19, 22, 24, 29, 32]. However, these methods generally require commissioning measurements in a water-equivalent phantom and many manual analysis that are time consuming. This means that a higher level of automation and more rapid quantitative analysis is desirable to accomplish full integration into daily clinical routine. Methods based on a direct correlation between the transit signals and doses in solid water phantom seem to be rapid and valid to convert EPID readings into doses in some points inside the patients treated by static beams. However, the most common EPIDs use the amorphous silicon (a-Si) as an indirect detector [1] that presents a response dependent on the change of the low-energy X-ray component [13]. This component can change with the distance from the central axis [20] with the field size and the interaction with the multileaf collimators (MLC) [11]. Due to these latter problems, the pretreatment verification of IMRT beams, carried out by EPIDs, can be obtained by applying a separate EPID response calibration to the open and the MLC transmitted beam fractions [30]. This work reports the results about the EPID response at the transmitted photons through the MLC in dynamic movement to obtain IMRT beams. A dose reconstruction algorithm for IMRT beams was formulated taking into account the corrections for both the EPID response (due to the presence of MLC on the beam central axis) and for the degree of inhomogeneity fluence of the beams with respect to the static beams. A software package connected with the Record–Verify system of the center was realized to reconstruct the isocenter dose to compare with the planned one.

## Materials and methods

### Linac unit

In this work, three Varian linacs, two operating at the Università Cattolica Sacro Cuore of Rome (UCSC) and one at the Centro di Riferimento Oncologico della Basilicata (CROB) of Rionero, that supply dynamic IMRT photon beams of 6 MV have been examined. The X-ray beams were delivered using a 120 leaf MLC with a minimum leaf separation gap of about 0.2 mm between the two opposing leaf banks. The monitor unit (MU) rate clinically used and here investigated is 400 MU/min.

Dose calibrations [9] were performed with an ionization chamber PTW (PTW Freiburg Germany) Farmer type model TM31010 (0.6 cm^3^). Table 1 reports the linac models equipped with the more recent aSi-EPID, model aS-1000. The 6 MV X-ray beams were characterized by TPR_20,10_ index ranging between 0.665 and 0.670.

**Table 1:** Varian linac models used in this work, including the image detector unit (IDU), the image acquisition system (IAS) and the EPID arm model

Linac Varian (institution)	IDU	IAS	EPID arm
2300 DXH (UCSC)	aS1000	3	Exact
2100 C/D (UCSC)	aS1000	3	R-arm
Trilogy (CROB)	aS1000	3	Exact

### Treatment planning system

Eclipse (Varian Medical System) version 8.9 is the treatment planning system (TPS) used in both centers. The dose calculation was performed using the AAA algorithm, with a dose grid resolution of 2.5 mm. The transmission factor and dosimetric leaf separation gap were measured in water phantom at 10 cm depth as indicated by Varian TPS Manual. These data were used by TPS to account for the transmitted radiation through the MLC. The computed dose at the source-axis distance (SAD) coincident with the isocenter point of radiotherapy treatments is here named *D*_iso,TPS_.

A dynamic IMRT beam is technically structured as a sequence of several elementary segments (in theory up to 320), with photon fluence delivery occurring also during the movement of leafs between two consecutive segments.

In this paper, simulated intensity-modulated beams (IMRT) were used to formulate an algorithm for the isocenter dose reconstruction. Square IMRT beams of size *L* = 5, 10, 12, 17 cm were obtained by the Fluence Editor function supplied by the TPS Eclipse. In particular using an electronic brush, it is possible to change X-ray fluence in selected regions of the beam assigning a brush transmission factor (BTF). A BTF lesser than one means an attenuation of the fluence; on the contrary, an increase in the fluence is obtained with a BTF greater than one. In other words, the BTF determines the MLC-speed on the regions swept by the brush. At the end, the TPS supplies the MU for an assigned dose at a reference point in phantom.

The Digital Communication in Medicine (DICOM) RT-file supplied by TPSs provided MLC positions and the MU number of each sequence. These information were used by an in-house software developed in Matlab code (Math Works, Inc., Natich, MA, USA) to obtain a beam integral intensity map (in terms of MU with a pixel resolution of 2 × 2 mm^2^) useful for the computation of a fluence inhomogeneity index of the beam (Sect. 2.4.1).

In addition, the total number of MU delivered, MU_tot_, and those delivered with the MLC closed, MU_clo_, on the beam central axis were used to determine the ratio:

1 $${\text{MU}}_{\text{c}} = \frac{{{\text{MU}}_{\text{clo}} }}{{{\text{MU}}_{\text{tot}} }},$$

which was then used for EPID response correction (Sect. 2.3.2).

### EPID transit signals

#### aSi-EPID signals

The EPID systems (Table 1), described in detail elsewhere [18, 22, 29], include (1) an image detection unit (IDU) featuring the detector and accessory electronics; (2) an image acquisition system (IAS), interfacing the hardware that controls and reads the IDU aS1000; and (3) a dedicated workstation (Portal Vision PC). The aS1000 IDU worked with a matrix of 1024 × 768 pixels, with a resolution of 0.392 × 0.392 mm^2^ (0.246 × 0.246 mm^2^ at the SAD) and a total sensitive area of about 40 × 30 cm^2^. The frame acquisition time is 111 ms.

The source to EPID distance (SED) can vary between 100 and 170 cm; moreover, lateral and longitudinal movements of the panel are also possible. All the measurements reported in this work were obtained at the SED equal to 159 cm, as carried out in a previous work, whose objective was an in vivo dosimetry procedure for open beams [8].

The EPIDs were used in the integrate image acquisition mode using the Varian Vision software, version 7.3.10 SP3, for all measurements, i.e., the imaging starts with beam on and stops when the beam turns off. All the images were stored as two-dimensional grayscale images determined as the average over all the subframes collected during the irradiation. The final image was automatically corrected for individual pixel sensitivity, dead pixels and dark current by the acquisition software as reported in Van Esch’s paper [29]. The images were exported as DICOM files to be analyzed. In particular, by means of an in-house software, the rough pixel signals in terms of arbitrary unit (au) were subtracted from the number 16384 (2^14^) and multiplied by the number of subframes. The EPID characterization in terms of reproducibility and signal linearity with MUs is reported in a previous paper [8].

#### aSi-EPID signals in the presence of MLC on the beam

Wih respect to the static open beams, the presence of the MLC supplies (1) a preferential attenuation of lower-energy photons by the source and (2) a production of a photon-scattered component by MLC. This means that EPID response can depend on the photon spectrum variation.

The dependence of the EPID response on the MLC configuration for the three different devices used in this work was investigated irradiating solid water phantoms (SPs) (Gammex Middleton, Winsconsin 53562-0327, USA) of thicknesses *w* = 12, 22, 32, 42 and 52 cm, with square homogeneous fluence beams of size *L* = 5, 10, 12 and 17 cm. The field was obtained in static and in dynamic (dy) modalities; these were last obtained using rectangular segments with gaps equal to 0.5, 1, 4 cm and *L* in length. To obtain the same dose at the phantom mid-plane, *w*/2 (coincident with the SAD), for all beams, leaf velocities decreased as the segment dimension decreased.

An ion-chamber Farmer (PTW Freiburg Germany) model 30310, 0.6 cm^2^ in volume, positioned on the beam central axis at SP mid-plan (*w*/2) was used to measure the dose *D*(*w*/2, *L*) and *D*_dy_(*w*/2, *L*) in terms of cGy/MU for static and dy-beams, respectively. All these measurements were carried out applying the IAEA international code [9]. Moreover, the transit signals for both static beams, *s*_*t*_ (*w*,*L*), and dy-beams, *s*_*t*,dy_(*w*,*L*), in terms of au/MU were obtained. This way, the ratios, *k*_dy_, could be defined as follows:

2 $$k_{\text{dy}} (w,L,{\text{MU}}_{\text{c}} ) = \frac{{\left[ {s_{t} \left( {w,L} \right)/D\left( {w/2, \, L} \right)} \right]}}{{\left[ {s_{{t,{\text{dy}}}} \left( {w,L} \right)/D_{\text{dy}} \left( {w/2, \, L} \right)} \right]_{{{\text{MU}}_{\text{c}} }} }},$$

where MU_c_ is the fraction of the delivered MU with the MLC closed on the beams central axis (Sect. 2.2).

### Transit dosimetry

#### Simulated IMRT beams

**Fig. 1:**
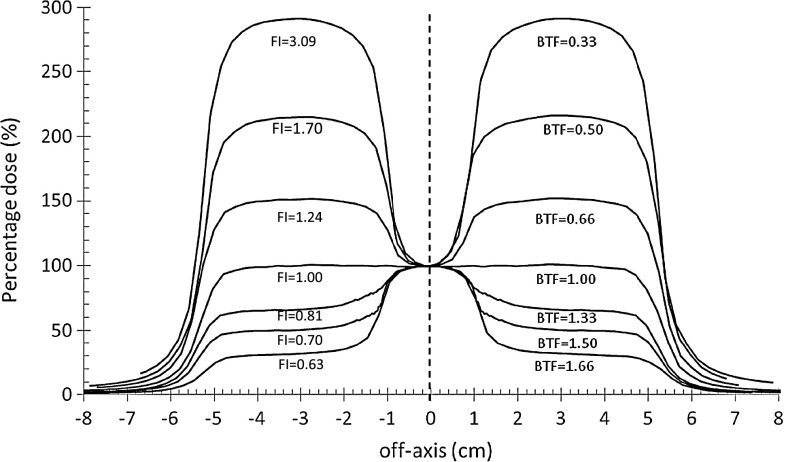
Percentage dose profiles along the principal axis of a square beam 10 × 10 cm^2^ across the fluence inhomogeneity direction of the simulated 6MV IMRT beams obtained with some BTF values. The IMRT beams supplied the same dose values at the SAD coincident with the SP mid-plane, with *w* = 22 cm. The indexes FI are reported too

The approach the authors have been pursuing over the years is to generate look up factors to convert transit signal by EPIDs into dose values on the beam central axis [21]. The method is based on the correlation ratios between the EPID transit signals and the doses at the isocenter point of SPs. The recent results [8, 23] are obtained for the 3D-CRT beams encouraged for the application in IMRT to develop a IVD procedure for Varian dynamic IMRT beams, based on a generalized algorithm. Correlation functions have been obtained irradiating SPs of different thicknesses, *w* = 12, 22, 32, 42, 52 cm by a set of square simulated IMRT beams with *L* = 5, 10, 12 and 17 cm at the SAD (Sect. 2.2). The beams presented a rectangular central area where the fluence was modified with respect to the rest of the field. In particular, the IMRT beams showed an homogeneous fluence on a square field, exception for the fluence of the central area, *L* × 2 cm, modified by BTF values equal to 0.05, 0.10, 0.20, 0.33, 0.50, 0.66, 1.33, 1.50 and 1.66. So, 36 IMRT beams were realized. Figure 1 shows the percentage dose profiles along the direction where the fluence inhomogeneity varies, obtained by the TPS at mid-plane SP with *w* = 22 cm. For each beam, the MUs were determined by the TPS for the same dose values at the SAD coincident with the SP mid-plane.

The external boundaries of an integral IMRT beam are similar enough to those of conformed beams used for the 3D-CRT, and the difference is in the fluence homogeneity. The software in Matlab developed to obtain an integral intensity map in terms of MU (Sect. 2.2) supplied also the computation of a fluence inhomogeneity, FI, index defined as

3 $${\text{FI}} = \frac{{\sum\nolimits_{i = 1}^{N} {{\text{MU}}_{i} \cdot a_{i} } }}{A} \cdot \frac{1}{{\overline{\text{MU}}_{\text{c}} }}$$

where the MU_*i*_ are the monitor units of the N pixels, each one of area *a*_*i*_ = 2 × 2 mm^2^; A is the total surface within the external boundaries of the IMRT beam determined multiplying the single area, *a*_*i*_, by *N*; and $\overline{\text{MU}}_{\text{c}}$ is the mean value of the MUs obtained by 9 pixels centered on the beam central axis, in other words, the mean value within an area of 6 × 6 mm^2^. Relative X-ray fluences for clinical IMRT beams were measured by a PTW seven29 2D array [5] and used to compute the FI indices by Eq. 3. The data were within 5 % equal to the FI indices determined using the MU intensity maps.

#### Ion-chamber and EPID measurements using simulated IMRT beams

It is well recognized that for typical IMRT fields (where the departure from reference conditions is dominant), the dose measurement by ion-chambers can be affected in minimal part by the changes of the water/air stopping power ratios, as a function of beam energy, and mostly by the specific type of the ion-chamber [2, 4, 26]. So, accepting an additional uncertainty for ion-chambers absolute dose determinations of IMRT fields, specific ion-chambers are recommended for pretreatment verifications. Following these indications and using the simulated IMRT beams, the SP mid-plane doses *D*(*w*/2, *L*, FI) in terms of cGy/MU (at the SAD) were obtained by a Seminflex PTW ion-chamber, model 31010 (0.125 cm^3^ in volume) [7], positioned along the beam central axis in the regions where the beams presented negligible dose gradients (Fig. 1). Positioning the SP with mid-plane at the SAD and the EPID at the SED, the transit signals in terms of au/MU, *s*_*t*_(*w*, *L*, FI), corrected by the factor, *k*_dy_ (Eq. 2), were obtained averaging the s(x,y) values of 24 × 24 central pixels (corresponding to an area of 6 × 6 mm^2^ at the SAD, fitting enough the ion-chamber internal dimensions, 5.5 mm in diameter and 6.5 mm in length). This way using the same SPs used in Sect. 2.3.2, the correlation ratios between the transit signals and the mid-plane doses at the SAD were obtained by:

4 $${\text{F}}_{\text{IMRT}} (w,L,{\text{FI}}) = \frac{{s_{t} (w,L,{\text{FI}}) \cdot k_{\text{dy}} }}{{D(w/2,L,{\text{FI}})}}$$

The ratios between F_IMRT_ (*w*,*L*,FI) (Eq. 4) and the *F*(*w*,*L*) obtained for open beams in the same experimental conditions [8] supplied the factors:

5 $$k_{F} (w,L,{\text{FI}}) = \frac{{F_{\text{IMRT}} (w,L,{\text{FI}})}}{F(w,L)}$$

In order to take into account the changes of scattered photon contributions to EPID due to different EPID to phantom distance, a new set of transit signal measurements were carried out shifting the mid-plane phantom to distances *d* = ±1, ±3 and ±5 cm from the SAD. This way irradiating the SP with the IMRT beams, we could obtain the following ratios, *f*_IMRT_(*w*, *L*, *d*, FI), between the transit signals:

6 $$f_{\text{IMRT}} (w,L,d,{\text{FI}}) = \frac{{s_{t} (w,L,{\text{FI}})}}{{s_{t} (w,L,d,{\text{FI}})}}$$

Finally, as already done with Eqs. 4 and 5, the factors:

7 $$k_{f} (w,L,d,{\text{FI}}) = \frac{{f_{\text{IMRT}} (w,L,d,{\text{FI}})}}{f(w,L,d)}$$

were determined, where the *f*(*w*,*L*,*d*) were obtained for open beams in the same experimental conditions [8].

In this work, the tissue maximum ratios (TMR) [3] for intensity-modulated beams (TMRM) have been defined by the ratios:

8 $${\text{TMRM}}(\delta ,L,{\text{FI}}) = D(\delta ,L,{\text{FI}})/D(\delta_{\hbox{max} } ,L,{\text{FI}})$$

The measurements were carried out by the Seminflex PTW ion-chamber, model 31010 positioned in SP, on the beam central axis and irradiated with 6MV IMRT simulated beams, characterized by the index FI. In particular, the ratios were obtained by the doses measured at depths *δ* and *δ*_max_ (the maximum dose depth) both at the SAD. The data by Eq. 8 were fitted versus *δ* and used to determine the ratio:

9 $${\text{TMRM}}(L,{\text{FI}})_{w/2}^{{w_{\text{iso}} }} = D(w_{\text{iso}} ,L,{\text{FI}})/D(w/2,L,{\text{FI}})$$

with both depths, *w*_iso_, and *w*/2, at the SAD.

### *D*_iso_ reconstruction

By considering the algorithm for 3D-CRT *D*_iso_ reconstruction [8], an equation suitable for IMRT beams was formulated as:

10 $$D_{\text{iso}} = S_{t} \cdot k_{\text{dy}} \left[ {\frac{{f(w,L,d) \cdot k_{f} (w,L,d,{\text{FI}})}}{{F(w,L) \cdot k_{F} (w,L,{\text{FI}})}}{\text{TMRM}}(L,{\text{FI}})_{w/2}^{{w_{\text{iso}} }} } \right]$$

where *S*_*t*_ (in terms of au) is the integral transit signal obtained averaging the signals of 8 × 8 central pixels (named macro-pixels, about 2.0 × 2.0 mm^2^ at the SAD), while all other parameters have been previously defined.

Propagating in quadrature the uncertainties of the parameters in Eq. 10 (obtained by the fits) and the uncertainty of *D*_iso,TPS_ [22], the acceptance criteria for the ratio

11 $$R = \frac{{D_{\text{iso}} }}{{D_{{{\text{iso}},{\text{TPS}}}} }}$$

has been determined equal to 0.950 ≤ *R* ≤1.050.

A software package to perform IVD procedures, connected with the Record–Verify system of the center, was developed. It consists of two integrated modules. The first one uses DICOM files from CT scanner and TPS to determine the parameters reported in brackets of Eq. 10. In particular, CT scan data are used to measure, along the beam central axis, the phantom thickness, z, and the isocenter depth, *d*_iso_. The respective water-equivalent thicknesses, *w*, and *w*_iso_ were determined as the product of z and *d*_iso_ with the physical density, obtained by the linear relation between the electronic and physical density. For each beam, by TPS DICOM-RT files, dosimetric data, as the MU and *D*_iso,TPS_, and the geometrical data, as the field shape, were obtained and stored. The fraction MU_c_ (Eq. 1) was used to determine the correction factor *k*_dy_ (Eq. 2).

FI computation (Sect. 2.4.1) is included in this first DISO module, as well as the computation of the equivalent square field size, *L*, according to the conventional Sterling [28] equation *L* = 2XY/(X + Y), where for rectangular static fields, *L* is calculated considering X as the jaw or MLC bank distance and Y as defined by the jaw settings. In a previous work [21], the equivalent square field size L for 3DCRT beams was determined using, as X, the mean of all apertures defined by opposing leaves within the field and, as Y, the distance corresponding to the open leaves (width of the leaves multiplied by the number of unclosed leaves). For the IMRT beams, *L* was determined in the same way [17]. In conclusion, all the parameters reported in brackets in Eq. 10 could be acquired for every IMRT beam in about 30 s.

The second DISO module was developed to obtain the ratio *R* (Eq. 11) and to present the IVD results on a computer screen.

#### Test in phantom

Thirty-six IMRT clinical beams planned for 15 pelvic (3 patients, 5 fields each) and 21 for head–neck tumors (3 patients, 7 fields each) were delivered with the gantry at 0° in a SP 22 cm thick, positioned with the distance d (*w*_iso_ − *w*/2) equal to 0, ±3, ±5 cm. In particular, the *D*_iso_ value for each beam was computed twice; the first value was obtained by Eq. 10 and the second one by the same equation where *k*_*f*_ and *k*_*F*_ were assumed equal to one and the TMR values [3] were used in place of the TMRM. By these two *D*_iso_ values, the ratios *R* were computed to verify the consistence of the factors reported in the bracket of the Eq. 10.

The sensitivity of the method to intercept mistakes in patients thickness was also analyzed irradiating phantoms with different dimensions. Indeed, the changes of anatomical patient dimension during the treatment are one of the major cause of dosimetric changes during IMRT treatments.

#### IVD tests

Five patients, treated with 7 clinical IMRT beams for head–neck cancer, were tested by the IVD method. The IMRT beams were delivered at the gantry angles at 0°, 51°, 103°, 154°, 206°, 257° and 309°. The patients in supine position were immobilized by a mask and doses between 50.4 and 66.0 Gy were planned. A pretreatment verification of all the IMRT beams was carried out only once before the fractioned treatments, using the Portal Dosimetry, version 8.8 (Varian Medical System).

For each patient, before the first treatment section, two square megavoltage beams were used at 0° and 90° gantry angles to obtain images to compare with the corresponding digitally reconstructed radiographs (DRR) by the TPS. Once reached a satisfying overlapping between the square megavoltage and the DRR images, the mask of patient was then marked by tattoos. The latter were used to reproduce the patient setup in the successive fractions. We verified that this method assured the reproducibility of bone landmarks within ±3 mm along the three spatial directions.

Taking into account this level of setup reproducibility, *D*_iso_ was determined using both the average signal of the central macro-pixels (2 × 2 mm^2^) and the average signals of other eight macro-pixels (with identical size) around the central macro-pixels. Therefore, nine *R* ratios were determined and the *R* value nearer to one was selected in the region of about ±3 mm (at the SAD) around the beam central axis.

This way each of the 35 clinical IMRT beams was tested 10 times obtaining 350 tests in terms of *R* ratios, in order to verify the agreement within the acceptance criteria equal to 0.950 ≤ *R* ≤1.050.

## Results

### *D*_iso_ reconstructions

**Fig. 2:**
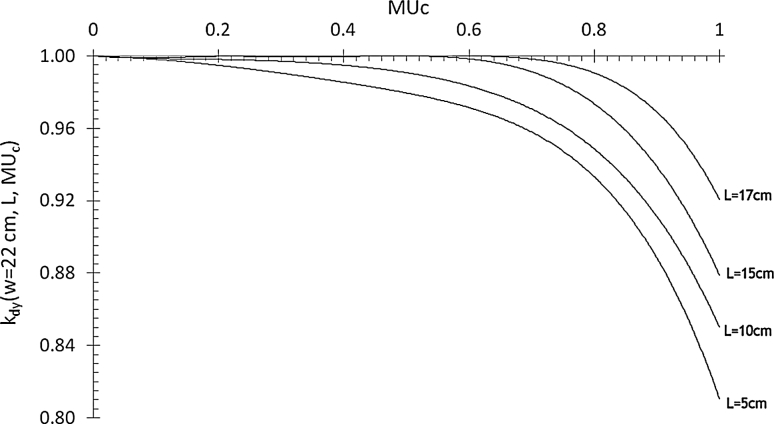
*k*_dy_(*w* = 22 cm, *L*, MU_c_) averaged values obtained by the three aS1000 EPIDs, using a SP with *w* = 22 cm and different dynamic field sizes, *L*

Using square uniform beam, delivered in dynamic MLC modality, the *k*_dy_(*w*,*L*,MU_c_) ratios (Eq. 2) were obtained averaging the values determined by the three EPIDs. The differences between the ratios obtained with the three EPIDs were within the experimental errors. Figure 2 shows the data fit obtained irradiating a SP 22 cm thick. It is possible to observe that the correction increases when the MU_c_ fraction approaches values near to one. Moreover, increasing the field size *L*, the segment-gap dimension increases to obtain the same fraction MU_c_. This justifies the results in Fig. 2, where, for fixed values of MU_c_, the EPID overresponse decreases due to the minor contribution to the X-ray component from the MLC closed for large fields. Finally, the correction increases with SP thickness, indicating an increase in the low-energy X-rays component, probably responsible for the overresponse of the silicon detectors [11, 30].

**Fig. 3:**
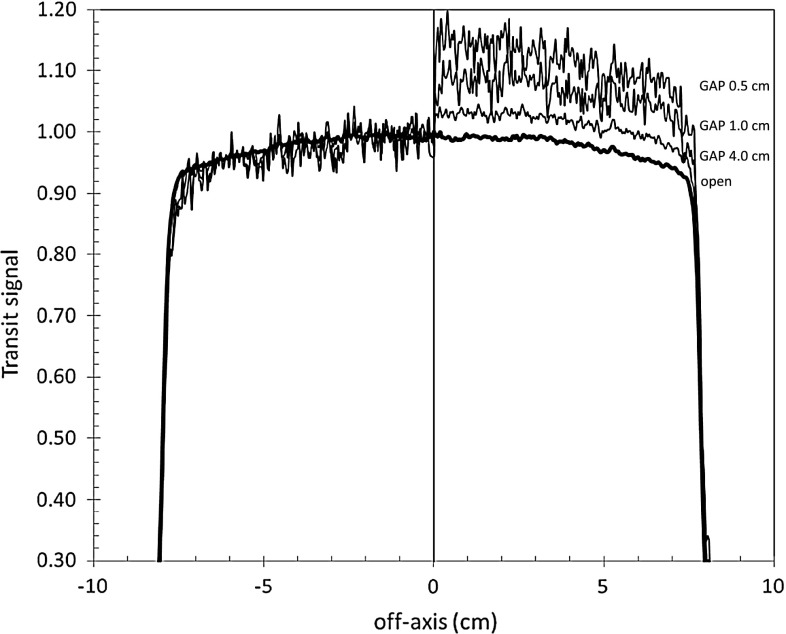
EPID transit signal profiles (at the SED) obtained irradiating a SP (*w* = 22 cm) with four square fields 10 × 10 cm^2^. On the right side the hemi-profiles obtained by an open field and dynamic beams (obtained with 3 segments of *L* = 10 cm length and different gaps) are reported. On the left side, the same hemi-profiles corrected for the *k*_dy_ factors, are reported

Figure 3 reports EPID transit signal profiles along the principal axis where the leaves travel. In particular, three 10 × 10 cm^2^ dynamic beams and one open beam (all supplying the same dose at phantom mid-plane) were used to irradiate a SP (*w* = 22 cm). The dynamic beams were obtained using segment gaps equal to 0.5, 1 and 4 cm that supplied MU_c_ fractions equal to 0.87, 0.91 and 0.97, respectively. On the right side of Fig. 3, the hemi-profiles exhibit large discrepancies. On the left side, instead, the hemi-profiles obtained multiplying the transit signal for the factors *k*_dy_(*w* = 22 cm, *L* = 10 cm, MU_c_) are in good agreement with the open beam profile.

**Fig. 4:**
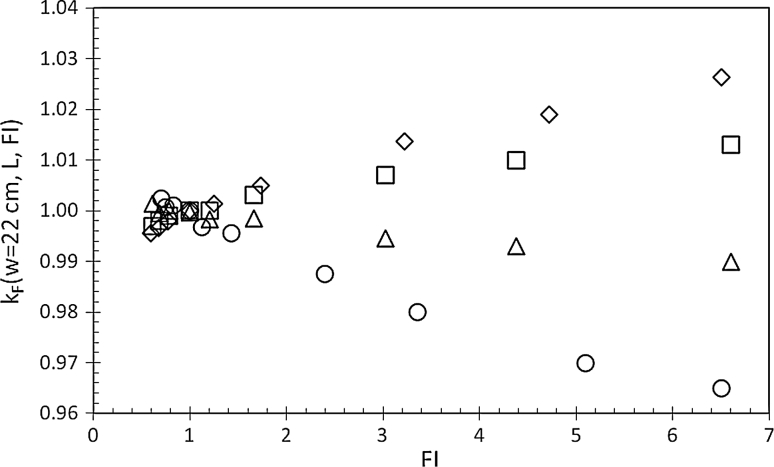
Ratios *k*_*F*_(*w* = 22 cm, *L*, FI) as a function of the FI index for 6MV IMRT beams with *L* = 17 cm (*open diamond*), *L* = 12 cm (*open square*), *L* = 10 cm (*open triangle*) and *L* = 5 cm (*open circle*)

The FI index of the simulated IMRT beams ranged between 0.5 and 6.5. Changes of *k*_*F*_(*w*,*L*, FI) within ±0.4 % (i.e., within the measurement reproducibility) were observed for similar beams delivered by the three linacs here used. Therefore, values shown in Fig. 4 were obtained averaging data collected from the three beams from the different linacs. In particular, Fig. 4 reports the ratios in Eq. 5 for some IMRT beams, with *L* = 5, 10, 12 and 17 cm. The changes observed for the factor *k*_*F*_(*w* = 22 cm, *L*, FI) as a function of FI index are the result of the different scattered photon contributions at the phantom mid-plane on EPID.

**Fig. 5:**
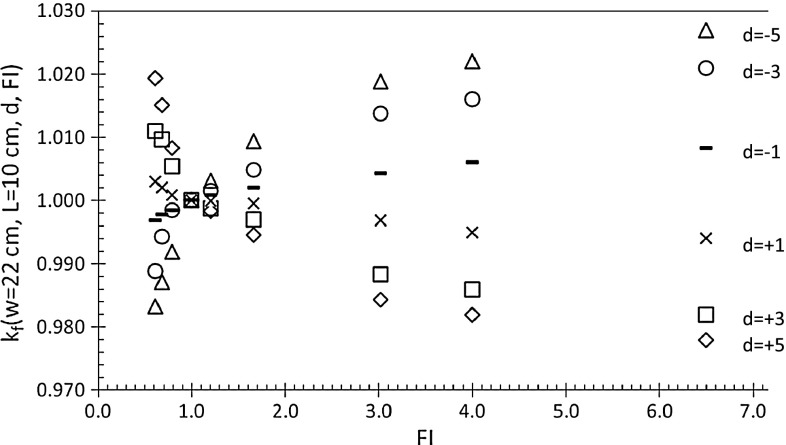
*k*_*f*_(*w* = 22 cm, *L* = 10 cm, *d*, FI) factors obtained for the 6MV IMRT beams for different *d* values

Figure 5 reports the *k*_*f*_(*w* = 22 cm, *L* = 10 cm, *d*, FI) ratios (Eq. 7) determined as a function of FI, ranging between 0.5 and 6.5. For d equal to ±1, ±3 and ±5 cm, the ratios varied within ±3 %. For the three beams, from the three different linacs, the ratios resulted the same, within the measurement reproducibility (±0.5 %), so data in Fig. 5 are the averaged values.

**Fig. 6:**
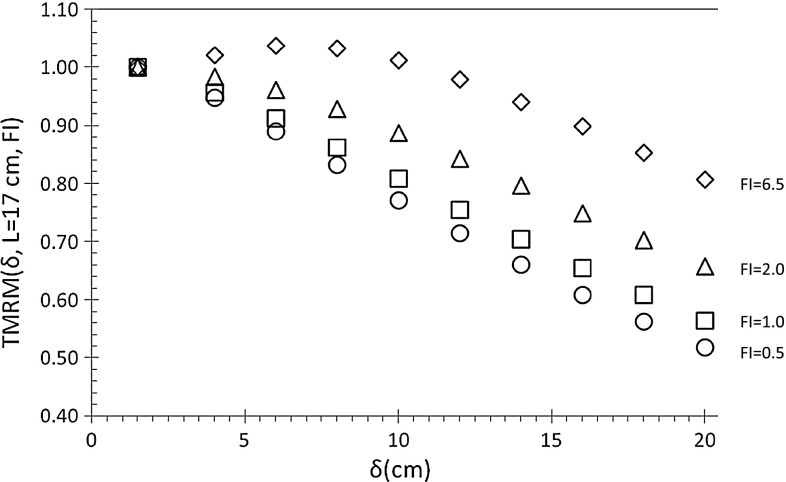
TMRM(*δ*, *L* = 17 cm, FI) ratios measured in SP (*w* = 42 cm) using simulated IMRT beams with *L* = 17 cm and FI = 0.5 (*open circle*), FI = 1.0 (*open square*), FI = 2.0 (*open triangle*), FI = 6.5 (*open diamond*)

Figure 6 shows the TMRM(δ, L = 17 cm, FI) (Eq. 8), measured in a SP (*w* = 42 cm) irradiated with simulated IMRT beams (*L* = 17 cm) and characterized by the FI index ranging between 0.5 and 6.5.

The ratio *k*_dy_ (Eq. 2), the factors *k*_*F*_ (Eq. 5) and *k*_*f*_ (Eq. 7) and the TMRM ratios (Eq. 8) were fitted by polynomial functions using a software package that minimize the sum of the residuals squares that are the differences between the experimental and the computed values [23].

In particular, the number of adjustable coefficients were chosen to obtain the data by the fit equation within the uncertainty of the experimental data.

### Clinical IMRT beams delivered in phantom

The FI indices of the clinical 36 IMRT beams ranged between 0.5 and 4.6. About 80 % of the beams presented a FI > 1.0, and their equivalent square size ranged between 8 and 16 cm.

**Fig. 7:**
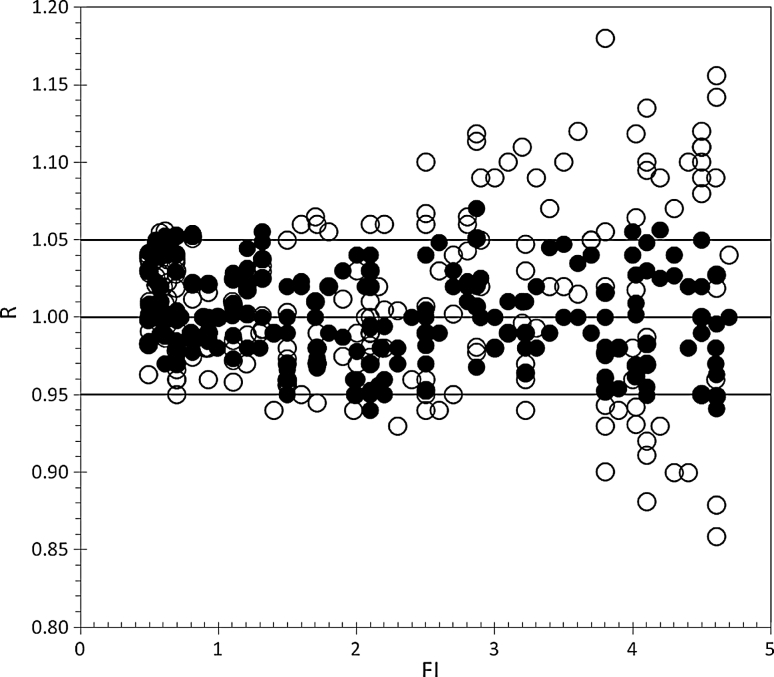
*R* ratios obtained for the dose reconstruction, *D*_iso_, in a SP with *w* = 22 cm, irradiated by 36 clinical IMRT beams. For each beam, 5 distances (*d* = 0, ±3, ±5 cm) were used. 180 data were obtained by the Eq. 10, (*filled circle*) taking into account the beam modulation characterized by the index FI. Open symbols (*open circle*) represents the same 180 ratios obtained again by Eq. 10 assuming *k*_*f*_ = *k*_*F*_ = 1 and TMR for *open fields*

*D*_iso_ was reconstructed in SP with *w* = 22 cm, at the depths, *w*_iso_, equal to 6 cm (*d* = −5 cm), 8 cm (*d* = −3 cm), 11 cm (*d* = 0), 14 cm (*d* = +3 cm) and 16 cm (*d* = +5 cm), shifting the SP. In particular, 180 R ratios (Sect. 2.5.1) were obtained with *D*_iso_ reconstructed using Eq. 10 (closed symbols) and other 180 ratios (open symbols) using the same equation taking into account (1) the k_dy_ factor, (2) a fluence homogeneity FI = 1 and (3) the TMR [3] for open beams. For the 36 IMRT beams, the correction factor, *k*_dy_, for the 36 IMRT beams ranged between 0.99 and 0.86. Figure 7 shows that for FI lower than 1.5, the results presented negligible differences, while for FI > 1.5, the differences were up to 15 %. The results show that the dose reconstruction by Eq. 10 supplied R ratios within the tolerance level of ±5 % and the average *R* ratio resulted equal to 1.002 ± 0.030 (2 SD). The introduction of artificial mistakes as the variation of phantom thickness showed that the procedure was able to intercept changes in dose reconstruction of about 5 %/cm, in agreement with the attenuation in water-equivalent medium for 6 MV X-ray beams.

### IVD tests for head–neck tumors

Five patients treated for head–neck tumors were tested by IVD. The FI index of the beams changed between 0.6 and 5.1. The results were displayed on a computer screen after 2 min from the end of the treatment; in particular for each beam, the *R* ratios (Sect. 2.5.2) of different tests were plotted on a diagram.

For each therapy fraction, the average value of the *R* ratios computed on the 7 fields ranged between 0.986 and 1.010, while the *R* ratios obtained for all the 350 tests ranged between 0.945 and 1.037 with a mean value equal to 1.004 ± 0.040 (2 SD).

## Discussion

We have demonstrated the feasibility of an IVD method based on correlation functions to reconstruct *D*_iso_ for dynamic IMRT beams by means of a software connected to the Record–Verify system of the center.

This work reports the results about the change in EPID response due to the transit photon transmitted through the MLC for dynamic IMRT. Figure 3 shows that the correction factor, *k*_dy_, increase when the MU_c_ approaches to one and the phantom thickness increases. This is due to the presence of a low-energy component in the transmitted X-ray beam, coming from MLC and phantoms.

In this study, an index of fluence inhomogeneity, FI, has been formulated to characterize the dynamic IMRT beams. In the literature, a number of different indexes are reported; each of one was developed for the assessment of the complexity inherent IMRT treatment plans, aiming to quantitative comparisons between planning solutions and predictions about their feasibility in terms of evolution criteria and action levels [14, 31]. In this study, the FI index (Eq. 3) seems easy to determine in each center, because it is formulated using information about MLC positions and MU numbers of each segment, recorded in each DICOM-RT file. Moreover, the discrepancy (±5 %) obtained (Sect. 2.4.1) between the FI computation by the MU map and the experimental relative fluence was estimated to be negligible in the selection of the parameters in Eq. 10.

Thirty-six simulated IMRT beams, that presented a range of fluence inhomogeneity FI between 0.5 and 6.5 were used to develop a generalized algorithm (Eq. 10) that can be used for both 3D-CRT beams (using the factors *k*_dy_, *k*_*F*_ and *k*_*f*_ equal to one and the TMR) and dynamic IMRT beams, supplied by Varian linacs.

The procedure was tested determining *D*_iso_ in a SP irradiated with 36 clinical IMRT beams for pelvic and head–neck tumors, with a FI index ranging between 0.5 and 4.6. Equation 10 assured an agreement between *D*_iso_ and *D*_TPS_ within 5 %, with an average *R* ratio equal to 1.002 ± 0.030.

Preliminary results of the IVD procedure applied at 5 patients undergoing radiotherapy for head–neck tumors, with IMRT beams characterized by FI indices between 0.6 and 5.1, provide an average ratio *R* on 350 tests equal to 1.004 ± 0.040.

## Conclusions

The objectives of this study were two. The first one was the formulation of a IVD procedure with an easy commissioning for Varian linacs equipped with aS1000 EPID. Indeed, all the parameters in Eq. 10 can be derived by results obtained in a previous paper [8] and in the present work. In particular, the characterization of the IMRT beams in terms of index FI can be obtained easily by each TPS. The second aim was to reach an automatic analysis using the Record–Verify system to supply the *D*_iso_ check on a computer screen in quasi-real time at the end of the treatment. This way the checks may be performed by the same staff that irradiates the patient, obtaining direct information about the actual patient treatment.

On the basis of these results, the authors intend (1) to implement the method to other beam qualities (10 and 15 MV) and (2) adopting the γ-analysis [12] between the 2D-portal images obtained in different fractions. Even if this last is not a 2D dosimetric comparison, the overcoming of alert criteria can be a suitable option to intercept the causes of off-axis signal changes [6], maintaining the objective of real-time results.
